# Recurrent adult jejuno-jejunal intussusception due to inflammatory fibroid polyp – Vanek’s tumour: a case report

**DOI:** 10.1186/1746-1596-9-127

**Published:** 2014-06-27

**Authors:** Kenneth M Joyce, Peadar S Waters, Ronan M Waldron, Iqbal Khan, Zolt S Orosz, Tamas Németh, Kevin Barry

**Affiliations:** 1 Department of Surgery, Mayo General Hospital, Mayo, Ireland; 2 Department of Histopathology, Mayo General Hospital, Mayo, Ireland

**Keywords:** Vanek’s tumour, Intussusception

## Abstract

**Background:**

Adult intussusception is a rare but challenging condition. Preoperative diagnosis is frequently missed or delayed because of nonspecific or sub-acute symptoms.

**Case presentation:**

We present the case of a sixty-two year old gentleman who initially presented with pseudo-obstruction. Computerised tomography displayed a jejuno-jejunal intussusception, which was treated by primary laparoscopic reduction. The patient re-presented with acute small bowel obstruction two weeks later. He underwent a laparotomy showing recurrent intussusception and required a small bowel resection with primary anastomosis. Histological examination of the specimen revealed that the intussusception lead point was due to an inflammatory fibroid polyp (Vanek’s tumour) causing double invagination.

**Conclusions:**

Adult intussusception presents with a variety of acute, intermittent, and chronic symptoms, thus making its preoperative diagnosis difficult. Although computed tomography is useful in confirming an anatomical abnormality, final diagnosis requires histopathological analysis. Vanek’s tumours arising within the small bowel rarely present with obstruction or intussusception. The optimal surgical management of adult small bowel intussusception varies between reduction and resection. Reduction can be attempted in small bowel intussusceptions provided that the segment involved is viable and malignancy is not suspected.

**Virtual Slides:**

The virtual slide(s) for this article can be found here: http://www.diagnosticpathology.diagnomx.eu/vs/7292185123639943

## Background

Intussusception is the invagination of a segment of the gastrointestinal tract into an adjacent segment. Intussusception is rare in adults, accounting for one percent of all cases of intestinal obstruction and five percent of all intussusceptions [1]. In adults, intussusception is typically due a pathologic lead point within the bowel, which is malignant in over 50% of cases [2].

## Case presentation

**Figure 1:**
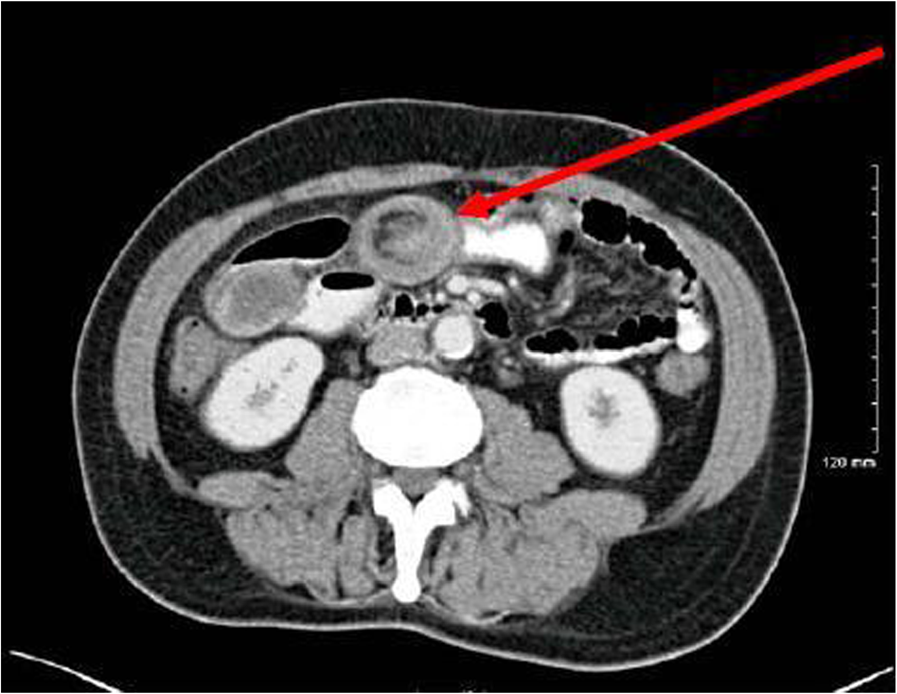
CT showing target lesion of jejunal intussusception (red arrow) – interpreted radiologically as benign.

**Figure 2:**
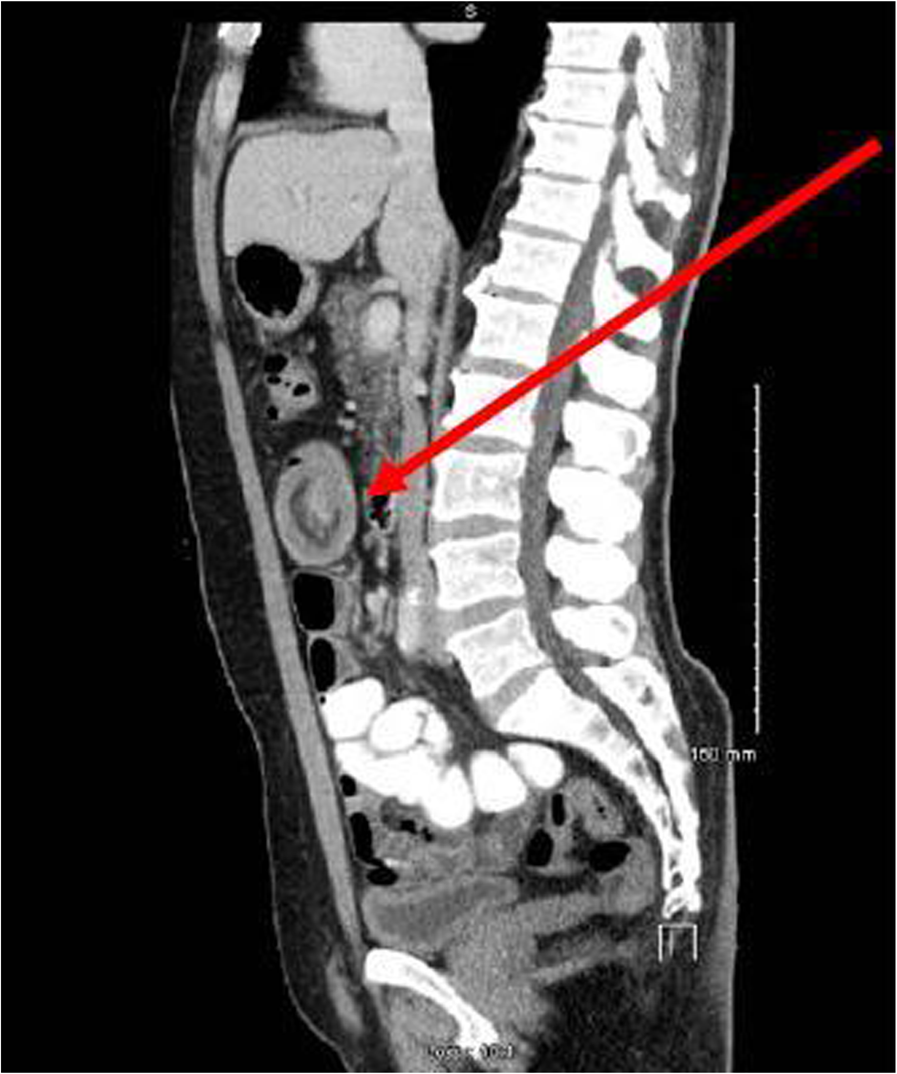
MRI showing jejunal intussusception (red arrow).

**Figure 3:**
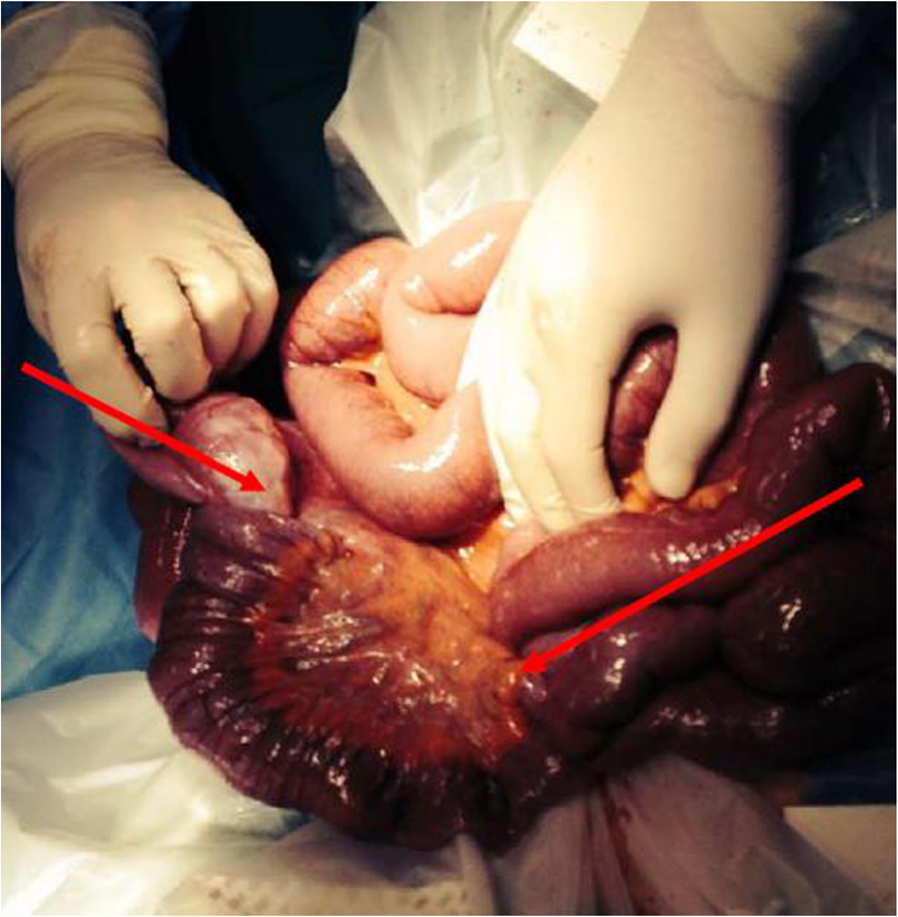
Intra-operative photograph of recurrent intussusception demonstrating double invagination (red arrows).

**Figure 4:**
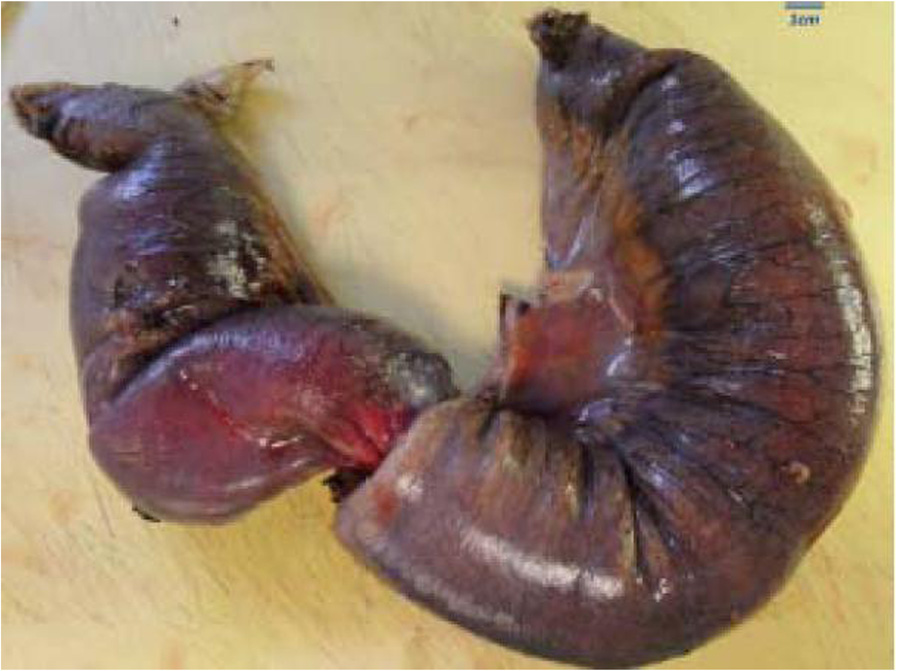
Resected jejunal intussusception (left side: intussusception, right side: intussuscipiens).

**Figure 5:**
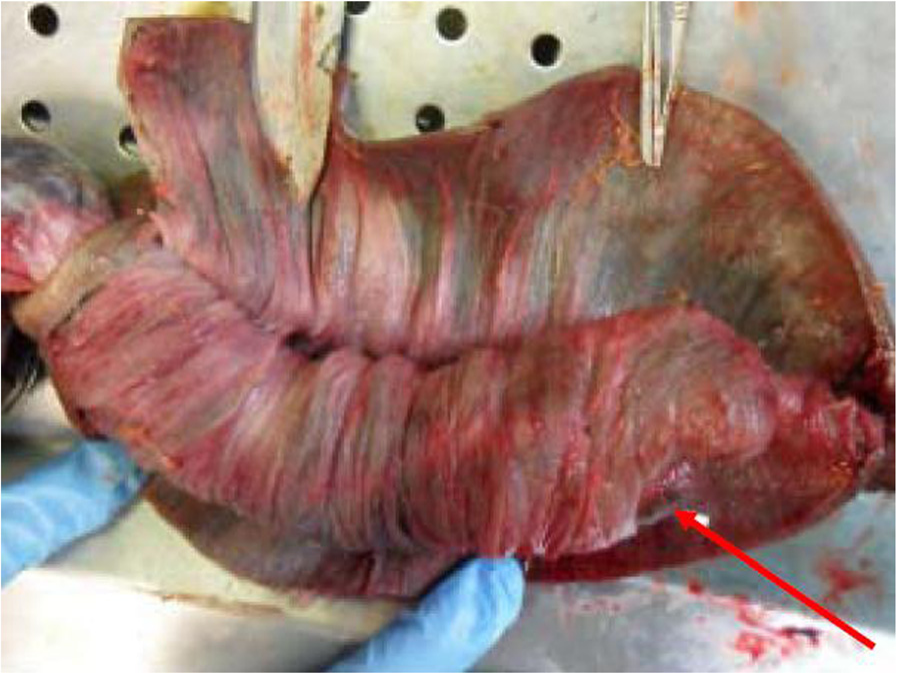
The invaginated area. The red arrowhead shows the ulcerated apical part of the polypoid tumour.

**Figure 6:**
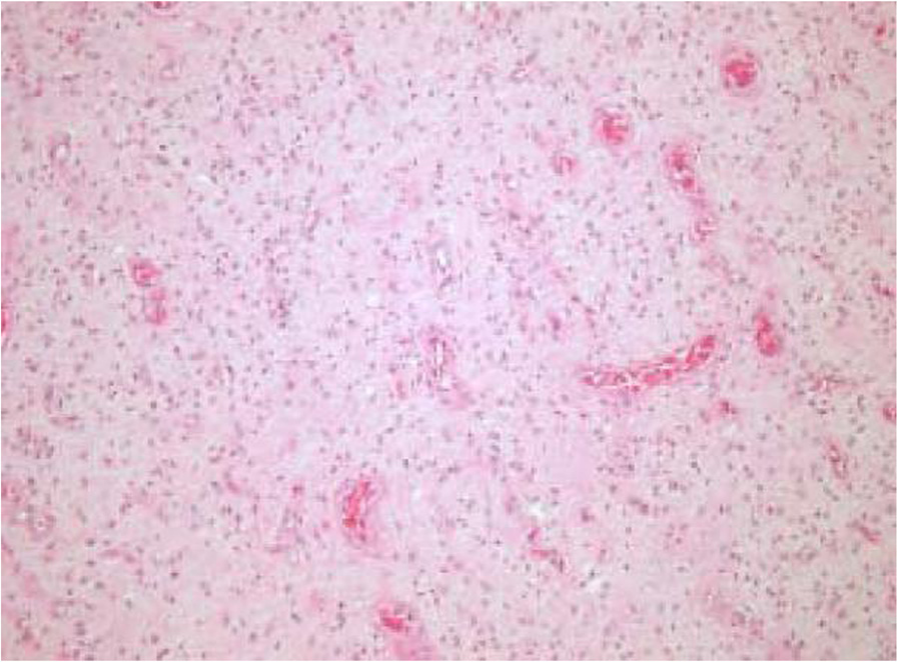
The tumour is composed of polygonal, stellate and spindle cells on a vascular background containing regular small to medium sized vessels (low power image).

**Figure 7:**
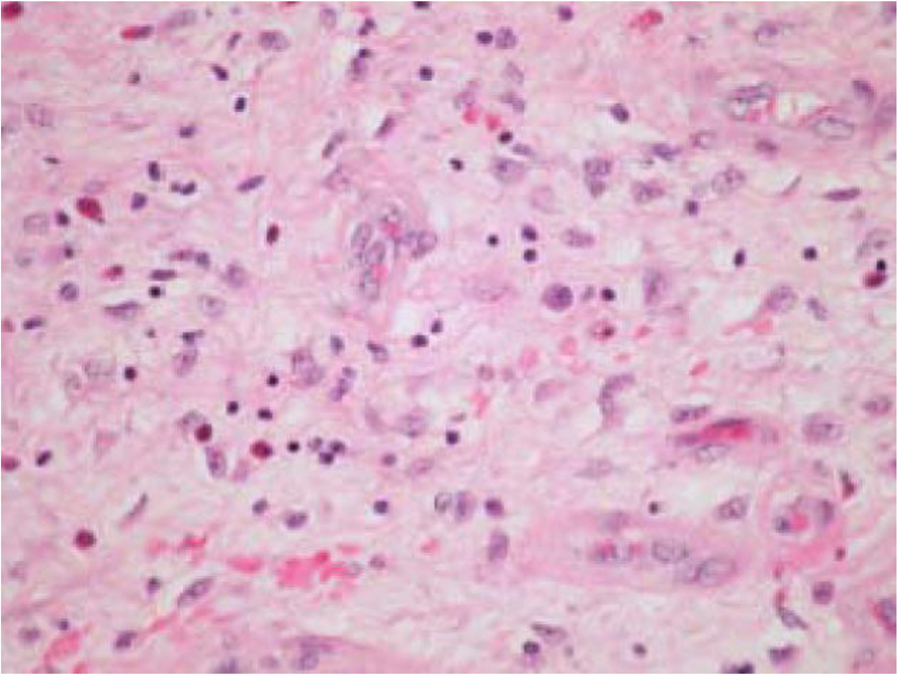
The tumour is dispersed by lymphocytes, eosinophils, histiocytes and plasma cells (high power image).

We present the case of a sixty-two year old gentleman who presented to our emergency department complaining of a 12 hour history of abdominal pain and distension. This was an acute onset central, crampy abdominal pain. He had associated anorexia but no vomiting. He denied any history of weight loss. His laboratory investigations were within normal limits. He had a coronary artery bypass graft performed four years previously and also had haemochromatosis.Computerized tomography of the abdomen demonstrated diffuse thickening of small bowel loops with a doughnut configuration. These findings were suggestive of small bowel intussusception, measuring 12 cm in length. There was a fat density at the centre of the intussusception radiologically consistent with a lipoma (Figure 1).He underwent a diagnostic laparoscopy which revealed an intussusception of the proximal jejunum. This was managed intra-operatively by manipulation and laparoscopic reduction without resection. Macroscopically there was no visible pathology in the form of a mass or lesion. Post-operatively the patient did well and was discharged on day three. An out-patient MRI of the small bowel was performed which showed a recurrent dynamic focus of intussusception of the jejunum with dilatation of proximal small bowel loops, although the patient remained asymptomatic (Figure 2).Two weeks after his initial surgery this man represented to the emergency department with vomiting and abdominal distension. Clinical examination was consistent with small bowel obstruction. He underwent a laparotomy which found recurrent jejuno-jejunal intussusception (Figure 3). This was excised and a primary end-to-end anastomosis was performed. The patient had an uncomplicated post-operative course and remains well.Histological examination of the resected specimen revealed the presence of an inflammatory fibroid polyp (Vanek’s tumour), which acted as the pathologic lead point for intussusception (Figure 4). The specimen was a 330 mm long segment of small bowel displaying complete invagination of a 145 mm long segment of it. There was a 42×25mm polypoid lesion at the lead point showing focal surface ulceration. The proximal 150 mm long segment was dilated (perimeter 105 mm). The tumour invaginated into a jejunal segment which had already invaginated. This phenomenon is known as double invagination (Figure 5).The tumour invaginated into a jejunal segment which had already invaginated (double invagination). It was a relatively well circumscribed ulcerated submucosal lesion showing variable cellularity, formed by spindle and stellate cells set in a fibro-myxoid background which is scattered by inflammatory cells. The inflammatory infiltrate was composed of mainly eosinophil leukocytes mixed with less number of histiocytes, plasma cells, neutrophil leukocytes and lymphocytes. Among the spindle cells small and medium sized blood vessels were also present containing thickened walled vessels too. Towards the ulcerated surface haemorrhage, granulation tissue pattern and more brisk inflammatory reaction were noted. There was no evidence of dysplasia or malignancy (Figures 6 and 7). The immunohistochemical study reveals no staining with Desmin, ER, PR, Synaptophysin and S100. The spindle and stellate cells are positive for Vimentin but negative for CD34, CD117 and SMA.

## Conclusions

Adult intussusception may present with a variety of acute, intermittent, or chronic symptoms, thus making its preoperative diagnosis difficult. The classic triad of intussusception consisting of abdominal pain, palpable sausage-shaped mass, and heme-positive stools is rarely present. The majority of studies confirm that computerized tomography (CT) is the most accurate diagnostic tool for preoperative diagnosis of intussusception. As Figure 1 shows, the CT findings characteristic of intussusception include the appearance of a bowel-within-bowel configuration with or without fat and mesenteric vessels incorporated. Pseudo-kidney or sausage appearance on longitudinal sections and target or bulls eye sign on transverse sections are other reliable radiological indicators of intussusception.

Between 70% and 90% of cases of intussusception requiring surgery have a specific identifiable ‘lead point’ such as a benign or malignant neoplasm [3]. Secondary intussusception is caused by organic lesions, such as inflammatory bowel disease, postoperative adhesions, Meckel’s diverticulum, benign and malignant lesions, metastatic neoplasms or even iatrogenically, due to the presence of intestinal tubes [1]. Recent studies using CT and magnetic resonance imaging scans have shown that small bowel intussusception can occur without a demonstrable pathological cause [4]. Transient intussusception is more common in the proximal small bowel where the peristaltic activity is normally greater [5].

Inflammatory fibroid polyp (also known as Vanek’s tumour) is an uncommon cause of adult intussusception. It is a rare, benign, solitary polypoid or sessile lesion which was first described by Vanek in 1949 as a “gastric submucosal granuloma with eosinophilia” [6]. Although the term inflammatory fibroid polyp was introduced by Helvig and Renier [7] to indicate the non-neoplastic nature of this lesion, recent studies suggest that these lesions should be considered as PDGFRA-driven benign neoplasms indicating that the term inflammatory fibroid tumour may be more appropriate [8,9]. It may develop in various parts of the gastro-intestinal tract but most commonly in the gastric antrum and the ileum. The majority of Vanek’s tumours are asymptomatic and discovered as incidental findings during endoscopy. Vanek’s tumours arising within the stomach produce symptoms of pyloric obstruction or anaemia while those within the small bowel may rarely present with obstruction or intussusception [10]. Although radiological investigations are useful in identifying the intussusception, the definitive diagnosis of small bowel Vanek’s tumour requires histological confirmation following operative resection.

Using modern imaging techniques to evaluate patients with various abdominal symptoms, there has been a twofold increase in the incidence of recognized intussusceptions secondary to idiopathic and incidentally detected intussusception [11]. Some authors suggest that intussusceptions that lack a pathologic cause of obstruction on CT are likely self-limiting and do not require operation [12]. Although incidental intussusceptions have become much more common, the majority of adult intussusception cases are still associated with a pathologic lead point which, in many patients, is malignant.

It is important to differentiate between small bowel and colonic intussusception. Adult colonic intussusception is associated with primary carcinoma in 65–70% of cases, while adult small bowel intussusceptions are secondary to a primary malignancy in only 30–35% of cases [13,14]. A common cause of benign intussusception is colonic polyps [3]. Adverse prognostic factors associated with colonic polyps include overexpression of regenerating gene Iα, α-methylacyl-coenzyme A racemase and p16 [15,16]. The benefit of reduction before resection is decreased length of resected bowel. In cases of malignancy, concern exists that a reduction manoeuvre may cause the underlying tumour to spread. Several recent large series of adult intussusception have advocated en bloc resection without initial reduction in order to remove an involved colonic segment because most large bowel intussusceptions are malignant whereas most small bowel intussusceptions are benign [17]. The fundamental surgical consideration is to distinguish between benign and malignant lesions preoperatively. Pre-operative imaging in this case suggested that a lipoma was the cause of the jejunal intussusception and therefore a trial of laparoscopic reduction was initially performed.

Most surgeons accept that adult intussusception requires surgical intervention because of the large proportion of structural anomalies and the high incidence of associated malignancy. However, the extent and timing of bowel resection and the manipulation of the intussuscepted bowel segment during reduction remains controversial. When a preoperative diagnosis of a benign lesion is safely established, the surgeon may reduce the intussusception by milking it out in a distal to proximal direction. This may be carried out using laparoscopy in suitable cases. Reduction should not be attempted if there are signs of inflammation or ischemia of the bowel wall. Primary malignant neoplasms are more commonly found in colocolonic and ileocolic intussusceptions, and hence, resection is well-advised.

The optimal surgical management of adult small bowel intussusception varies between reduction and resection. Reduction can be attempted in small bowel intussusceptions provided that the segment involved is viable and malignancy is not suspected.

## Consent

Written informed consent was obtained from the patient for publication of this case report and case series and accompanying images. A copy of the written consent is available for review by the Editor-in-Chief of this journal on request.

## Competing interests

The author’s declare that they have no competing interests.

## Authors’ contributions

KJ and PS were involved in writing the paper. RW processed the clinical photographs. IK was the operating surgeon. ZO and TN were involved in analysing the specimen and producing the pathologic images. KB is the senior author and performed the final revision of the paper. All authors read and approved the final manuscript.
